# Plants regenerated from tissue culture contain stable epigenome changes in rice

**DOI:** 10.7554/eLife.00354

**Published:** 2013-03-19

**Authors:** Hume Stroud, Bo Ding, Stacey A Simon, Suhua Feng, Maria Bellizzi, Matteo Pellegrini, Guo-Liang Wang, Blake C Meyers, Steven E Jacobsen

**Affiliations:** 1 Department of Molecular, Cell and Developmental Biology, University of California, Los Angeles, Los Angeles, United States; 2 Department of Plant Pathology, Ohio State University, Columbus, United States; 3 Department of Plant and Soil Sciences, Delaware Biotechnology Institute, University of Delaware, Newark, United States; 4 Howard Hughes Medical Institute, University of California, Los Angeles, Los Angeles, United States; University of Cambridge, United Kingdom

**Keywords:** Rice, DNA methylation, tissue culture, small RNA, regeneration, Other

## Abstract

Most transgenic crops are produced through tissue culture. The impact of utilizing such methods on the plant epigenome is poorly understood. Here we generated whole-genome, single-nucleotide resolution maps of DNA methylation in several regenerated rice lines. We found that all tested regenerated plants had significant losses of methylation compared to non-regenerated plants. Loss of methylation was largely stable across generations, and certain sites in the genome were particularly susceptible to loss of methylation. Loss of methylation at promoters was associated with deregulated expression of protein-coding genes. Analyses of callus and untransformed plants regenerated from callus indicated that loss of methylation is stochastically induced at the tissue culture step. These changes in methylation may explain a component of somaclonal variation, a phenomenon in which plants derived from tissue culture manifest phenotypic variability.

**DOI:** http://dx.doi.org/10.7554/eLife.00354.001

## eLife digest

Rice is one of the most important food crops and is estimated to provide more than a fifth of the calories consumed by the world's population. For several decades, rice has been modified by conventional breeding methods to produce plants with increased yields and greater resistance to pests and harsh weather conditions. Efforts are also being made to create rice plants with superior yield traits and resistance to biotic and abiotic stresses using genetic engineering techniques.

Genetically modified plants are usually produced using tissue culture. New genes are introduced into plant cells that are growing in a dish, and each cell then replicates to form a mass of genetically identical cells. The application of plant hormones triggers the tissue to produce roots and shoots, giving rise to plantlet clones.

In addition to the genes that comprise its genome, the genetic make-up of an organism also includes its epigenome—a collection of chemical modifications that influence whether or not a given gene is expressed as a protein. The addition of methyl groups to specific sequences within the DNA, for example, acts as an epigenetic signal to reduce the transcription, and thus expression, of the genes concerned.

Now, Stroud et al. reveal that the techniques used to modify a plant's genome—in particular, the process of tissue culture—also affect its epigenome. They prepared high-resolution maps of DNA methylation in several regenerated rice lines, and found that regenerated plants produced in culture showed less methylation than control plants. The changes were relatively over-represented around the promoter sequences of genes—regions of DNA that act as binding sites for the enzymes that transcribe DNA into RNA—and were accompanied by changes in gene expression. Crucially, the plants' descendants frequently also inherited the changes in methylation status. These results are likely part of the explanation for a phenomenon called somaclonal variation, first observed before the era of modern biotechnology, in which plants regenerated from tissue culture sometimes show heritable alterations in the phenotype of the plant.

**DOI:** http://dx.doi.org/10.7554/eLife.00354.002

## Introduction

Rice is one of the world's most important food crops, and genetic modifications are extensively used for various purposes such as to increase yield and tolerate harsh environments. Tissue culture has been heavily used for decades for transformation procedures to generate transgenic crops such as rice and maize (Rao et al., 2009). A previous study has reported that Arabidopsis cell suspension culture has a different epigenomic profile compared to wild-type plants, such that certain transposable elements (TEs) become hypomethylated and certain genes become hypermethylated (Tanurdzic et al., 2008). This raised the question of how tissue culture processes affect the epigenome of regenerated plants derived from tissue culture. Changes in the epigenome have been proposed to be a source of somaclonal variation (i.e., phenotypic variation among regenerated plants) for decades (Kaeppler and Phillips, 1993; Kaeppler et al., 2000; Thorpe, 2006; Rhee et al., 2010; Miguel and Marum, 2011; Neelakandan and Wang, 2012). Indeed, some evidence suggesting changes in the epigenome of regenerated plants have been reported at several specific loci or by methods such as methylation sensitive restrictive enzyme digestion (Neelakandan and Wang, 2012). However, the extent of methylation changes on a genome-wide level has not been previously assessed. Because, unlike most crops, Arabidopsis is almost exclusively transformed via Agrobacterium-mediated floral dip methods that do not utilize tissue culture (Clough and Bent, 1998), Arabidopsis is not a good model for the study of the effect of plant regeneration on the epigenome. The study of the model plant rice, however, may have practical implications for other crop species that are transformed using similar tissue culture methods.

The rice genome is DNA methylated in all three cytosine contexts (CG, CHG, CHH, where H=A, T, or C), with high levels of CG and CHG methylation and very low levels of CHH methylation (Feng et al., 2010; Zemach et al., 2010). Whole genome bisulfite sequencing (BS-seq) enables measurement of DNA methylation at single nucleotide resolution and thus allows one to distinguish DNA methylation in different cytosine contexts (Cokus et al., 2008; Lister et al., 2008).

To investigate the effect that tissue culture processes have on regenerated rice epigenomes, we generated genome-wide, single-nucleotide maps of DNA methylation in several regenerated rice lines that had been transformed with various transgenes, callus, and rice regenerated from tissue culture without transformation. We observed that the tissue culture procedure induced stable changes in DNA methylation in regenerated plants, such that all regenerated lines had ectopic losses of DNA methylation. We found that loss of DNA methylation occurred stochastically, affecting individual plants somewhat differently, was associated with loss of small RNAs, and changes were enriched at promoters of genes. Loss of DNA methylation at promoters was associated with altered expression of particular genes.

## Results

We performed deep BS-seq to map DNA methylation in nine regenerated rice lines in the Nipponbare ecotype background that were transformed by various transgenes and were at various stages of inbreeding after transformation: rice blast resistance lines PiZ-t, PiZ-t-839 (a non-functional PiZ-t), Pi9, and an RNAi line for flowering time regulator Spin1 (Zhou et al., 2006; Vega-Sanchez et al., 2008; Table 1). For the PiZ-t line, both transgenic and non-transgenic T2 and T4 plants were available by genetic segregation of the PiZ-t transgene (Table 1). For comparison, we profiled an untransformed wild-type line, which was used to generate all the regenerated lines, WT2003 (sample 1). WT2003 was also inbred 5–7 generations to produce WT2007 (sample 2), and WT2007 was inbred 5–7 additional generations to produce WT2011 (sample 3). We obtained an average genome coverage of 15× and error rates were low at 1.5%, 1.2%, 0.8%, for CG, CHG, CHH methylation, respectively, indicating high quality data (Table 1).

**Table 1.** BS-Seq samples analyzed in this study **DOI:** http://dx.doi.org/10.7554/eLife.00354.003

Sample	Description	Raw reads	Uniquely mapping reads	Coverage (X)	CG error rate	CHG error rate	CHH error rate
1	WT2003	231568902	100572780	13.5178	0.0176	0.0122	0.0099
2	WT2007	203541357	104376988	14.0292	0.0107	0.0087	0.0082
3	WT2011	187803109	84301904	11.3309	0.0158	0.0095	0.0065
4	T2-PiZt-11-R	229650259	118710094	15.9557	0.0139	0.0099	0.0069
5	T2-PiZt-11-S	263329602	136471411	18.3429	0.0101	0.0096	0.0076
6	T4-PiZt-11-R	270670871	131056700	17.6151	0.0117	0.0100	0.0074
7	T4-PiZt-11-S	252150298	128467721	17.2672	0.0096	0.0076	0.0074
8	T6-PiZt-11-R	237280137	121966745	16.3934	0.0105	0.0096	0.0064
9	T6-Pi9-R	204752699	86995742	11.6930	0.0106	0.0093	0.0050
10	T6-Spin1i-1-R	215451022	90468236	12.1597	0.0113	0.0088	0.0061
11	T2-PiZt-839-8-R (non functional PiZt)	238730281	117471332	15.7892	0.0129	0.0079	0.0056
12	T2-PiZt-839-8-S (non functional PiZt)	211006119	106172872	14.2705	0.0178	0.0129	0.0095
13	WT Callus 1	217121522	96145279	12.9228	0.0185	0.0178	0.0070
14	WT Callus 2	199261493	82617643	11.1045	0.0232	0.0222	0.0084
15	WT regenerated from tissue culture 1	218008835	116367626	15.6408	0.0170	0.0155	0.0078
16	WT regenerated from tissue culture 2	225202113	97905142	13.1593	0.0262	0.0206	0.0093
17	WT regenerated from tissue culture 3	252306428	106544735	14.3205	0.0194	0.0160	0.0073
18	WT2011 (replicate)	253971827	118140062	15.8790	0.0172	0.0148	0.0086

Number of raw sequencing reads, number of uniquely mapping reads (post-removal of identical reads), genome coverage (rice genome size = 372 Mb), and error rates are listed. DNA methylation levels of the chloroplast genome were used to estimate error rates. Samples 1–12 and samples 13–18 were prepared separately. “R” and “S” correspond to plants that either contain the transgene (R) or plants in which the transgene was segregated away (S).

**Figure 1.**
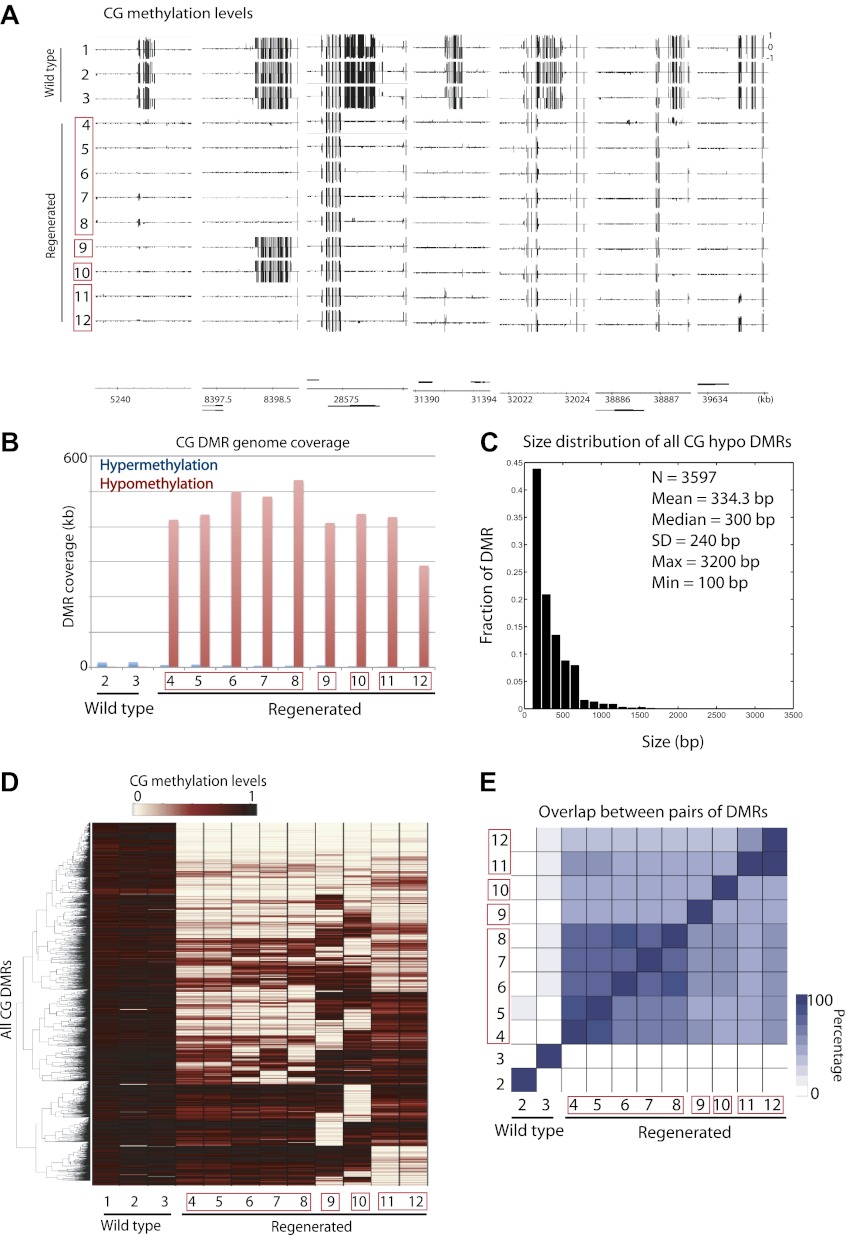
Aberrant loss of DNA methylation in regenerated rice. (**A**) Genome browser views of fractional CG methylation levels. Sample numbers correspond to those listed in Table 1. Regenerated samples of the same line are grouped together in red boxes. (**B**) Genome coverage of identified CG hypermethylation and hypomethylation DMRs. DMRs were defined relative to sample 1 (wild type). (**C**) Distribution of sizes of CG hypomethylation DMRs in regenerated plants. (**D**) Heat map representation of hierarchical clustering based on CG methylation levels within DMRs. Rows represent all 3610 CG-DMRs identified and columns represent the samples. (**E**) Overlap of CG-DMRs between samples. The bottom triangle represents the percent overlap of elements listed in the x-axis with those listed in the y-axis. The upper triangle on the other hand represents the percent overlap of elements listed in the y-axis with those listed in the x-axis. **DOI:** http://dx.doi.org/10.7554/eLife.00354.004

We observed strong losses of DNA methylation at certain sites in the genome in the regenerated plants but not in wild-type plants (Figure 1A). To further characterize these sites, we defined differentially methylated regions (DMRs) in CG contexts by applying stringent thresholds (see ‘Materials and methods'). We found that all regenerated plants tested were significantly enriched with CG hypomethylation DMRs (Figure 1B). On average, we identified 1344 CG hypomethylation DMRs in the regenerated plants, whose sizes ranged from 100 to 3200 bp (Figure 1C), whereas on average we identified only eight CG hypomethylation DMRs in the inbred wild-type lines (Figure 1—source data 1). Importantly, we observed hypomethylation even in the T2/T4 non-transgenic plants in which the transgenes had been segregated away (samples 5, 7 and 12), suggesting that loss of DNA methylation is likely due to the tissue culture or transformation process, but not due to the fact that the plants contain transgenes. While loss of DNA methylation in different regenerated lines did not always occur at the same sites (Figure 1D), there were significant overlaps of hypomethylation DMRs among regenerated lines (Figure 1E). This suggests that certain sites in the genome are susceptible to loss of DNA methylation in regenerated plants.

**Figure 2.**
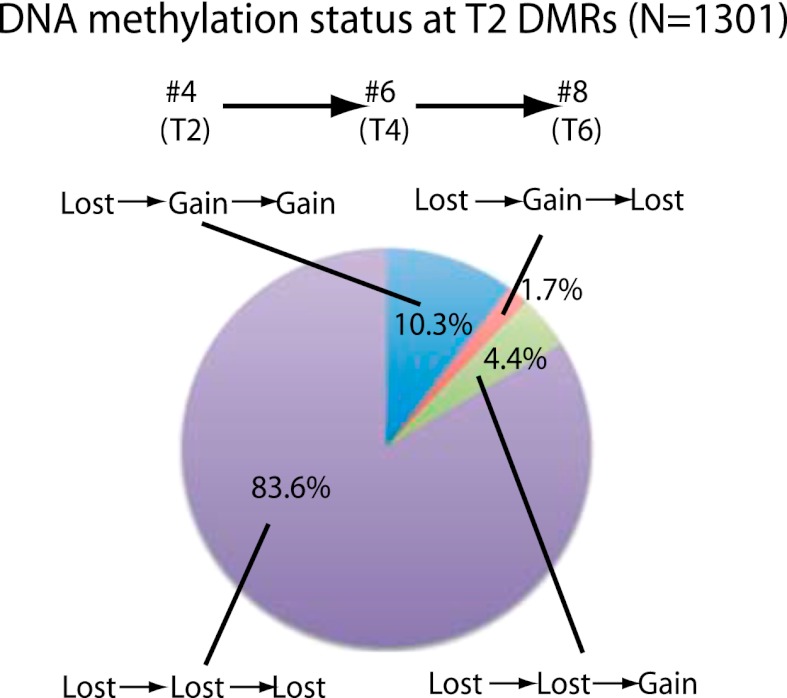
Stability of loss of DNA methylation over generations. Methylation status of sample 4 (T2) DMRs in T4 and T6 generations are indicated. Loss: less than half of respective wild-type CG methylation levels. Gain: more than half of respective wild-type CG methylation levels. **DOI:** http://dx.doi.org/10.7554/eLife.00354.006

We next investigated the stability of DNA methylation losses across generations. To test this, we analyzed a line for which we had plants in T2, T4, and T6 generations (samples 4, 6, 8). 84% of sites that lost CG methylation in the T2 did not recover methylation in the T4 and T6 generations (Figure 2). This suggests that most sites do not regain DNA methylation over several subsequent generations during the process of inbreeding. Approximately 10% of sites recovered methylation in T4, and this methylation was maintained in T6. In addition, 4.4% of sites recovered methylation in T6 but not in T4. This suggests that certain sites are able to regain methylation over generations. Approximately 2% of sites regained methylation in T4, but methylation was lost again in T6, suggesting that a small fraction of sites are epigenetically unstable and continue to switch states. Our results suggest that most of the DNA hypomethylation in regenerated plants was stable over generations.

**Figure 3.**
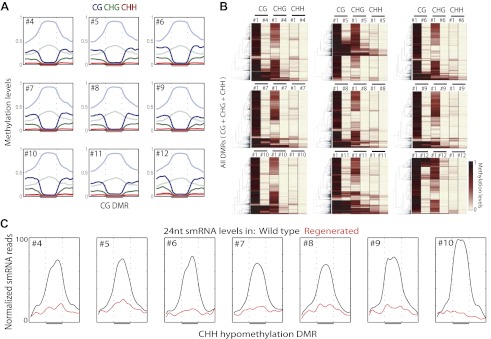
Loss of DNA methylation occurs in all three cytosine contexts. (**A**) Average distributions of DNA methylation in wild type (faded) and regenerated plants (solid) were plotted over defined CG hypomethylation DMRs in the indicated samples. Flanking regions are the same lengths as the middle region. (**B**) Heat map of DNA methylation levels within all defined hypomethylation DMRs (CG + CHG + CHH). (**C**) Average distribution of smRNA-seq reads in wild type (black) and regenerated plants (red) over defined CHH hypomethylation DMRs in indicated samples. Flanking regions are the same lengths as the middle region. **DOI:** http://dx.doi.org/10.7554/eLife.00354.007

Loss of DNA methylation in regenerated plants also occurred in non-CG contexts (Figure 1—source data 1). Loss of CG methylation was generally associated with loss of CHG methylation and to a lesser extent with loss of CHH methylation (Figure 3A,B). Small interfering RNAs of 24-nt in length (24-nt siRNAs) are associated with DNA methylation, and are required to guide CHH methylation to particular sites (Law and Jacobsen, 2010). We performed small RNA sequencing (smRNA-seq) on seven randomly chosen regenerated plants along with wild type (Table 2). We examined the distribution of 24-nt siRNAs over CHH hypomethylation DMRs and found that siRNAs are enriched over these sites in wild type, but eliminated in regenerated plants (Figure 3C). Hence loss of DNA methylation is associated with loss of 24 nt siRNAs. Moreover, these siRNA alterations independently confirm our findings showing loss of epigenetic marks at these loci.

**Table 2.** smRNA-seq samples analyzed in this study **DOI:** http://dx.doi.org/10.7554/eLife.00354.008

Sample	Description	Raw reads	Uniquely mapping reads
1	WT2003	22030663	3186666
2	WT2007	17069498	2598780
3	WT2011	14860767	2399713
4	T2-PiZt-11-R	22024881	3965317
5	T2-PiZt-11-S	17641623	3127938
6	T4-PiZt-11-R	18999415	3090933
7	T4-PiZt-11-S	22115074	4258752
8	T6-PiZt-11-R	12995193	2044615
9	T6-Pi9-R	16700524	3114923
10	T6-Spin1i-1-R	17275813	2973100

Number of raw sequencing reads and number of uniquely mapping reads are listed.

**Figure 4.**
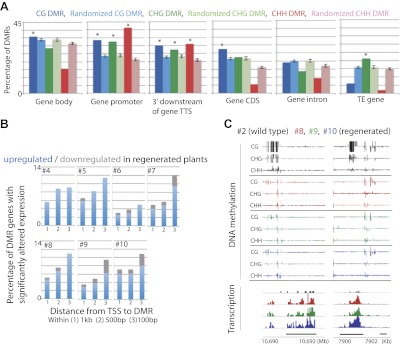
Loss of DNA methylation at promoters may impact gene expression. (**A**) Overlap of hypomethylation DMRs with indicated genomic elements. Observed overlap (dark bars) is compared to randomized regions of similar number and size distribution as the DMRs (light bars). Gene body: transcribed region of protein coding genes. Gene promoter: TSS to 2 kb upstream of TSS. 3' downstream of gene TTS (transcription termination site): TTS to 2 kb downstream of TTS. CDS: Coding sequence. TE: Transposable element. Error bars represent standard deviation. *Significant enrichment, p<0.01. (**B**) Percentages of genes with CG hypomethylation DMRs near TSSs that have significantly altered expression levels (fourfold up/down regulation, FDR<0.01). Genes with zero mRNA-seq reads in both wild type and regenerated samples were removed from the analyses. An average of 11.3 genes were deregulated. (**C**) Genome browser views of DNA methylation and gene expression levels. **DOI:** http://dx.doi.org/10.7554/eLife.00354.009

We next examined the genomic characteristics of sites that lost DNA methylation in regenerated plants. We tested the extent of overlap between 3597 CG DMRs, 1875 CHG DMRs, and 2298 CHH DMRs defined in the regenerated lines within gene bodies, gene promoters, downstream regions of genes, gene coding sequences, gene introns, and TE genes. Although loss of DNA methylation occurred at a variety of sites, we found the most significant enrichments of DMRs at the promoters of genes (Figure 4A). These genes were not significantly associated with any particular biological processes (data not shown). Rather, they appeared to be a random set of genes involved in different processes (Figure 4—source data 1). Recent studies in Arabidopsis have shown that spontaneous changes in methylation over generations predominantly occurred in gene bodies (Becker et al., 2011; Schmitz et al., 2011). It is possible that hypomethylation observed in regenerated plants occurs through an accelerated process of whatever mechanism causes spontaneous methylation changes over generations. Alternatively, since the DNA methylation changes we observed in regenerated plants was enriched in gene promoters, and was primarily in the direction of methylation loss, it could be a distinct phenomenon from the spontaneously occurring methylation changes in wild type.

While the losses of DNA methylation in regenerated plants occurred within a relatively small proportion of the rice genome, they were concentrated near protein-coding gene promoters and therefore in regions of the genome that are more prone to alter gene expression. We therefore examined the impact of hypomethylation on gene expression by performing mRNA-seq on the same seven randomly chosen regenerated plants as well as on wild-type plants (Table 3). We found that loss of DNA methylation at promoters was associated with higher expression levels of certain genes (Figure 4B,C, Figure 4—source data 1, Figure 4—figure supplement 1, 2). Notably, the closer the hypomethylation was to the gene transcription start site, the more likely the gene tended to be misregulated (Figure 4B). Furthermore, the expression of these genes was much more frequently increased, rather than decreased, suggesting that the misexpression of these genes is likely a direct consequence of losses of DNA methylation (Figure 4B). Hence loss of DNA methylation in regenerated plants is associated with deregulated transcription of certain protein-coding genes.

**Table 3.** mRNA-seq samples analyzed in this study **DOI:** http://dx.doi.org/10.7554/eLife.00354.015

Sample	Description	Raw reads	Uniquely mapping reads
2	WT2007	44029089	29461162
3	WT2011	33997755	22657098
4	T2-PiZt-11-R	42550136	27839598
5	T2-PiZt-11-S	43173764	28688381
6	T4-PiZt-11-R	46624891	35826861
7	T4-PiZt-11-S	31729173	22667633
8	T6-PiZt-11-R	46624532	35335627
9	T6-Pi9-R	38978541	30623633
10	T6-Spin1i-1-R	42280235	32485204

Number of raw sequencing reads and number of uniquely mapping reads are listed.

**Figure 5.**
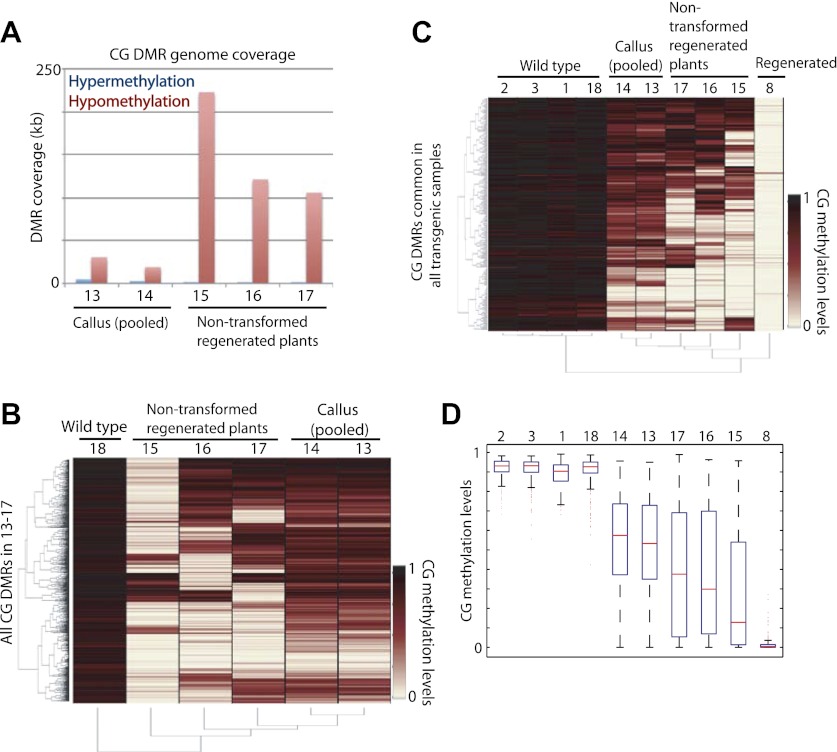
Tissue culture step induces loss of DNA methylation. (**A**) Genome coverage of identified CG hypermethylation and hypomethylation DMRs. DMRs were defined relative to sample 18 (wild type). (**B**) Heat map of CG methylation levels within all 1074 CG hypomethylation DMRs identified in samples 13 to 17 (callus samples and wild-type plants regenerated from callus). (**C**) Heat map of CG methylation levels within 241 CG hypomethylation DMRs that were observed in all tested regenerated plants. (**D**) Boxplot representations of (**C**). Red lines, median; edges of boxes, 25th (bottom) and 75th (top) percentiles; error bars, minimum and maximum points within 1.5 × IQR (Interquartile range); red dots, outliers. **DOI:** http://dx.doi.org/10.7554/eLife.00354.016

We further sought to determine whether it was the tissue culture process or the transformation process that induced loss of DNA methylation in regenerated plants. To test this, we performed BS-seq on callus and three individual plants regenerated from untransformed callus, all of which were derived from a single parent plant (WT2011; Table 1). We were not able to perform BS-seq on individual calli because calli at the stage of transformation did not yield enough genomic DNA. Instead, we pooled multiple calli, and sequenced two separate batches. We found a strong loss of DNA methylation in plants regenerated from untransformed callus (Figure 5A). Loss of DNA methylation in callus was much more modest, though significant (Figure 5A). This relatively weak loss of DNA methylation may be because individual calli lose DNA methylation at different sites (despite being derived from the same parent plant), and pooling multiple calli diluted the loss of DNA methylation. Consistent with this notion, individual plants regenerated from untransformed callus showed differences in sites that lost DNA methylation (Figure 5B). Furthermore, when examining methylation levels of these samples at CG hypomethylation DMRs that were common in all regenerated plants, we found significant losses of DNA methylation at these sites in callus (Figure 5C,D), indicating that the methylation losses observed in callus were at largely the same sites as those observed in regenerated plants. Like in the regenerated lines, the losses of DNA methylation in the non-transformed regenerated plants occurred stochastically, affecting DNA methylation in each plant somewhat differently (Figure 5A–D). In summary, the loss of DNA methylation in regenerated plants is likely caused by the tissue culture step, and not due to the transformation process.

**Figure 6.**
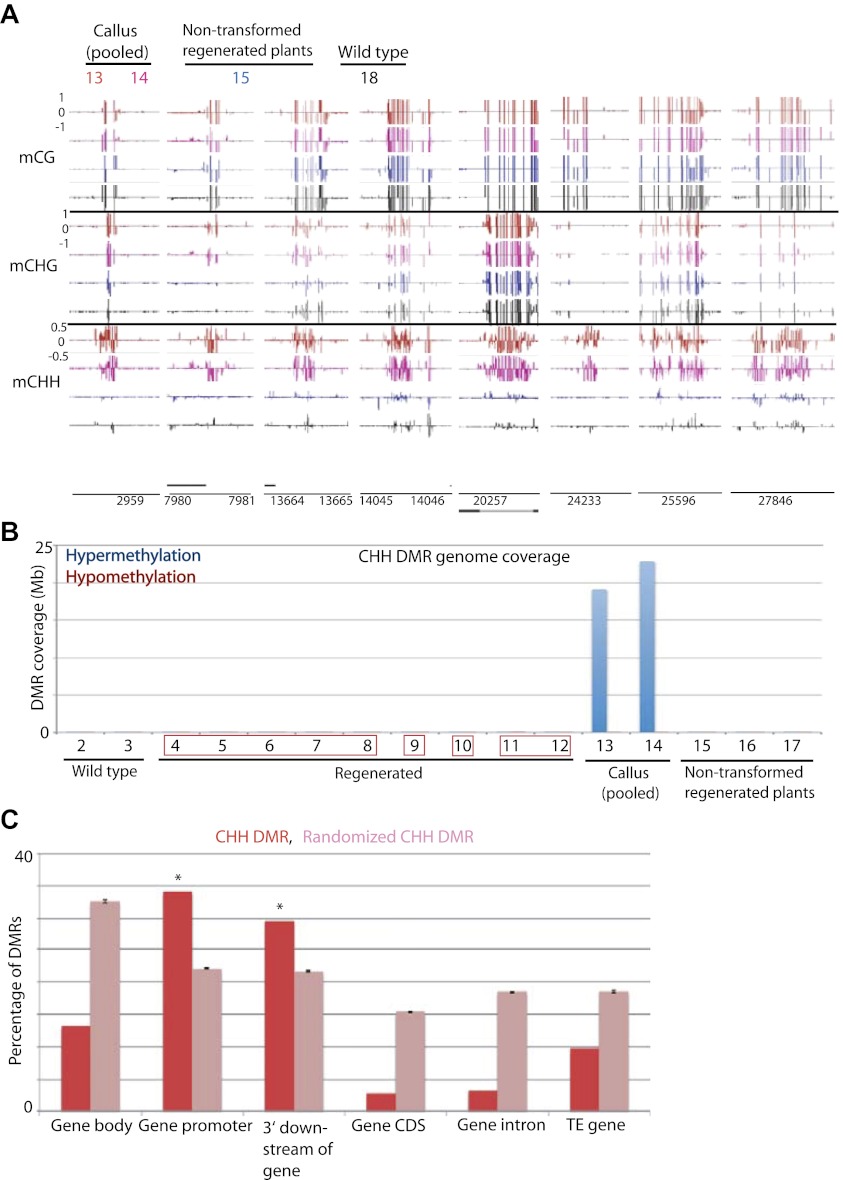
Tissue culture-induced CHH hypermethylation is eliminated upon regeneration. (**A**) Genome browser views of DNA methylation. (**B**) Genome coverage of identified CHH hypermethylation and hypomethylation DMRs. Regenerated samples of the same line are grouped together in red boxes. (**C**) Overlap of callus CHH hypermethylation DMRs with indicated genomic elements. Observed overlap (dark bars) is compared to randomized regions of a similar number and size distribution as the DMRs (light bars). Error bars represent standard deviation. **DOI:** http://dx.doi.org/10.7554/eLife.00354.017

Previous reports have indicated that certain genes are hypermethylated in Arabidopsis cell suspension culture and callus (Berdasco et al., 2008; Tanurdzic et al., 2008). Consistent with those data we found that rice callus showed hypermethylation throughout the genome (Figure 6A). Interestingly we found that the hypermethylation occurred specifically in CHH contexts (Figure 6A,B, Figure 6—figure supplement 1A), and showed high coincidence between the two callus samples (13 and 14) (Figure 6—figure supplement 1B). These CHH hypermethylated regions mostly corresponded to promoter regions (Figure 6C, Figure 6—figure supplement 1A). Hence in callus, certain promoters are CHH hypermethylated, while others are hypomethylated in all cytosine contexts. Interestingly, CHH hypermethylation observed in callus was completely lost in regenerated plants (Figure 6A,B, Figure 6—figure supplement 1A). This suggests that unlike tissue culture-induced DNA hypomethylation that is largely stable after regeneration, CHH hypermethylation is eliminated after regeneration.

**Figure 6—figure supplement 1.**
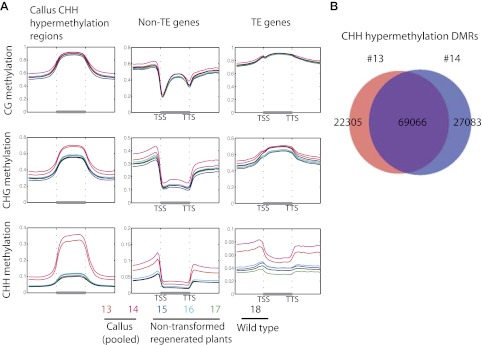
Callus induced CHH hypermethylation. (**A**) Average distribution of DNA methylation over defined CHH hypermethylated regions in callus, genes, and TE genes. Flanking regions are the same lengths as the middle region. (**B**) Overlap between defined CHH hypermethylation DMRs of the two callus samples in this study (13 and 14). **DOI:** http://dx.doi.org/10.7554/eLife.00354.018

## Discussion

In this report, we have investigated the effect that tissue culture processes have on the epigenome of regenerated plants by generating high-resolution maps of DNA methylation. Consistent with a previous study in Arabidopsis cell culture using microarray hybridization on chromosome 4 (Tanurdzic et al., 2008), we observed hypermethylation at certain genes in rice callus. We extend this observation by showing that hypermethylation predominantly occurs in CHH sequence contexts, most notably occurring at the promoters of genes. Interestingly, we found that this CHH hypermethylation was completely eliminated upon regeneration, suggesting that CHH hypermethylation may be linked specifically to the dedifferentiated state.

In contrast to Arabidopsis cell culture, we did not observe global hypomethylation at TEs in rice callus. Instead, we found that DNA methylation was specifically lost at certain sites in the genome, appearing to affect individual plants somewhat differently despite coming from the same parent plant. We found that loss of DNA methylation was maintained upon plant regeneration, and was largely stable over subsequent generations. It is possible that some of the DMRs affected only one homologous chromosome and were segregating. However, because we required DMRs to have at least 70% reduction in DNA methylation compared to wild-type, the sites we analyzed in Figure 2 are likely homozygous for loss of DNA methylation, consistent with their stability across generations. Loss of DNA methylation occurred in all sequence contexts, and was associated with loss of 24-nt siRNAs. Notably, these sites were frequently associated with promoters of genes, and loss of DNA methylation was associated with misregulation of expression of proximal protein-coding genes, indicating a biological importance of this phenomenon. Interestingly, genes significantly up-regulated (fourfold upregulated compared to wild type, p<0.01) in each regenerated line were somewhat different (Figure 4—figure supplement 3). For this reason, it is difficult to assess the severity of impact of misregulated gene expression for any particular regenerated line, since some lines may have more biologically important genes affected than others. This would correlate with the observation that somoclonal variation affects only a proportion of plants that arise from regeneration experiments (Kaeppler and Phillips, 1993; Kaeppler et al., 2000; Thorpe, 2006; Rhee et al., 2010; Miguel and Marum, 2011; Neelakandan and Wang, 2012).

Previous studies have shown that certain TEs such as *Tos17* and *mPing* are reactivated in tissue culture, and are associated with changes in DNA methylation (Neelakandan and Wang, 2012). While our results suggest that most DNA hypomethylation occurs near genes and are relatively depleted at TE related sequences (Figure 4A), some of the hypomethylation did occur proximal to TE genes (average of 62.1 TE genes per line). The association of loss of methylation with TE gene reactivation was not clear (data not shown), however very subtle depletion of DNA methylation was observed over reactivated TE genes (Figure 4—figure supplement 4), suggesting that loss of methylation may in part be responsible for reactivation of TEs.

Our results suggest that each regenerated plant has distinct DNA methylation profiles despite coming from the same parent (Figure 5A–D). It therefore appears that the tissue culture step induces DNA hypomethylation in a rather stochastic manner affecting individual plants differently. We further show that descendants of regenerated plants stably maintain most hypomethylation across plant generations (Figure 2). Indeed, lines derived from the same original regenerated plant show very similar methylation profiles (Figure 1E; samples 4–8 and 11–12). It has long been proposed that changes in the epigenome may be a source of somaclonal variation (Thorpe, 2006; Rhee et al., 2010; Miguel and Marum, 2011; Neelakandan and Wang, 2012). Our genome-wide data support this notion since we show that stochastic hypomethylation in individual regenerants is associated with misregulated expression of certain genes. These epigenetic changes likely explain a component of somaclonal variation that has been observed for decades in a number of plant species.

In summary, our results suggest that use of tissue culture leaves behind an epigenetic footprint in regenerated plants that is stable over multiple generations and may partially explain somaclonal variation. Whereas the material used in this study were self-fertilized plants, a common practice in the development of agricultural biotechnology traits is to introgress new transgene loci into commercial genetic backgrounds, meaning that the plants used in agriculture are many generations removed from the initial regenerated plants (Bregitzer et al., 2008; Bennetzen and Hake, 2009; Johnson, 2009; Yang et al., 2012). The crosses utilized in these introgression schemes are likely to correct the vast majority of tissue culture-induced epigenetic changes.

## Material and methods

### Rice material

Wild-type rice (*Oryza sativa* ssp *japonica* cv Nipponbare) and regenerated rice lines (in Nipponbare background) were used in this study (Zhou et al., 2006; Vega-Sanchez et al., 2008). Hygromycin was used as the selection marker in rice transformation. All the resistant plants were selfed for indicated generations (Table 1). Homozygosity was confirmed by PCR analysis of the transgene. Rice seeds were surface-sterilized and transferred to 1/2 MS medium. After germination, rice seedlings were transplanted into soil and kept in a growth chamber at 26/20°C under a 14-hr light/10-hr dark cycle. The rice plants regenerated from untransformed rice callus induced from Nipponbare seeds (WT2011) were prepared as previously described (Zhou et al., 2006; Vega-Sanchez et al., 2008). Rice leaf samples were collected at 3 weeks after transplanted into soil and the rice callus were harvested from the callus inducing media.

### Bisulfite sequencing (BS-seq) and analysis

BS-seq libraries were generated as previously described using premethylated adapters (Feng et al., 2011) using 1 μg of genomic DNA isolated using DNeasy Plant Maxi Kit (Qiagen, Hilden, Germany). Libraries were single-end sequenced on a HiSeq 2000, and reads were base-called using the standard Illumina software. The read counts for these libraries are listed in Table 1. Reads (50 nt) were mapped to the MSU 6.1 version genome using BS-seeker (Chen et al., 2010) allowing up to two mismatches. Identical reads were collapsed into one read. Fractional methylation levels were calculated by #C/(#C+#T).

DMRs for each sample were defined by comparing methylation levels to wild type in 100 bp bins across the genome. Fischer's exact test was used to identify bins that were significantly differentially methylated by comparing #C and #T (Benjamini*-*Hochberg corrected FDR < 0.01). In addition, we required an absolute methylation difference of 0.7, 0.5, 0.1, for CG, CHG, CHH methylation, respectively. Bins that were within 100 bp were merged. Finally, only bins that contained 10 informative cytosines (i.e., covered by ≥4 reads) in both the sample and wild type were considered as DMRs. Sample 1 was used for the wild type control for samples 2–12, whereas sample 18 was used for the wild type control for samples 13–17. This was because sample preparation (i.e., growth of plants and library constructions) were performed in two batches: 1∼12 and 13∼18.

All heat maps in this study were generated by complete linkage and using Euclidean distance as a distance measure. Rows with missing values were omitted for presentation purposes but did not affect the conclusions in the paper.

For determining overlap of DMRs with different genomic elements, we considered 1 bp overlap as an overlap. To assess significance, we generated 100 sets of ‘randomized DMRs' which mimicked both the number and size distributions as the observed DMRs, and examined their overlaps with the different genomic elements.

### mRNA/smRNA sequencing and analysis

RNA-seq libraries were constructed from total RNA isolated using TRIzol reagent (Invitrogen, Life Technologies, Carlsbad, CA) from leaf tissues of samples 2∼10. Total RNA (10 μg) for each sample was used to purify poly-A mRNA; this mRNA was used for synthesis and amplification of cDNA. The RNA-seq libraries were prepared using the TruSeq RNA Sample Preparation Kit from Illumina (San Diego, CA). Libraries were sequenced on an Illumina HiSeq 2000. The read counts for these libraries are listed in Table 3.

smRNA-seq libraries were constructed from total RNA isolated from the same tissues as described for the mRNA libraries, using the TruSeq Small RNA Sample Prep Kit from Illumina (San Diego, CA). The libraries were sequenced on the same Illumina HiSeq 2000 as the mRNA-seq libraries. The read counts for these libraries are listed in Table 2.

Gene annotations (MSU 6.1) were obtained from the Rice Genome Annotation Project website (http://rice.plantbiology.msu.edu/). mRNA-seq reads were mapped and processed as previously described (Stroud et al., 2012). Briefly, reads were uniquely mapped to the genome using Bowtie (Langmead et al., 2009) allowing two mismatches, and differentially expressed genes were defined by applying fourfold and FDR < 0.01 cutoffs. smRNA-seq reads were uniquely mapped to the genome using Bowtie allowing no mismatches, and reads were categorized by their lengths for analyses. Reads per kilobase per million mapped reads (RPKM) was used to quantify RNA-seq datasets.

### Accession numbers

All sequencing data have been deposited in the NCBI Gene Expression Omnibus with accession number GSE42410.

## Supplementary Material

10.7554/eLife.00354.005 Figure 1—source data 1. List of CG, CHG, CHH DMRs identified in this study. Defined hypomethylation DMRs for each sample are listed.
**DOI:**http://dx.doi.org/10.7554/eLife.00354.005

10.7554/eLife.00354.010 Figure 4—source data 1. List of genes with CG hypomethylation DMRs at promoters and their expression levels. Genes with CG hypomethylation DMRs at the promoter regions (TSS minus 2 kb to TSS) in samples 4–10 along with their normalized expression levels are listed. Also indicated are whether they were significantly up- or down-regulated based on fourfold and FDR < 0.01 cutoffs. Descriptions of genes were directly taken from the rice genome annotation project website (http://rice.plantbiology.msu.edu/).
**DOI:** http://dx.doi.org/10.7554/eLife.00354.010

## Figures and Tables

**Figure 4—figure supplement 1.**
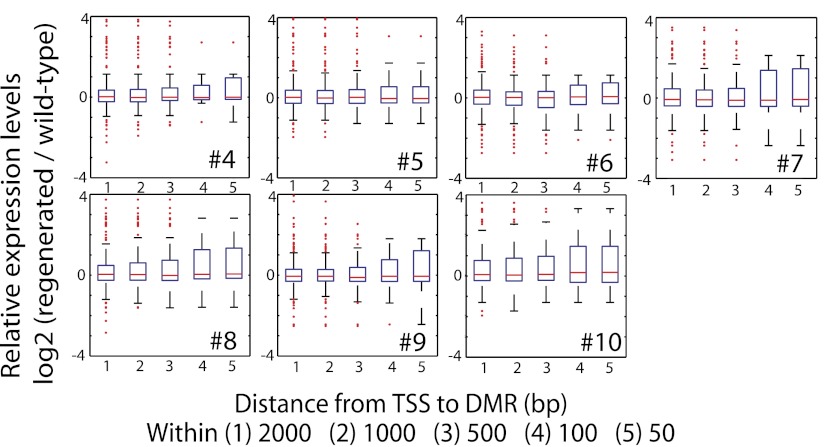
Impact of loss of DNA methylation at promoters on gene expression. Relative expression levels of genes with CG hypomethylation DMRs near TSS. Log2 ratios between RPKM values of indicated regenerated lines and wild type (sample 2) were calculated, and data is represented as boxplots. Genes with zero mRNA-seq reads in both wild type and regenerated samples were removed from the analyses. Red lines, median; edges of boxes, 25th (bottom) and 75th (top) percentiles; error bars, minimum and maximum points within 1.5 × IQR (Interquartile range); red dots, outliers. **DOI:** http://dx.doi.org/10.7554/eLife.00354.011

**Figure 4—figure supplement 2.**
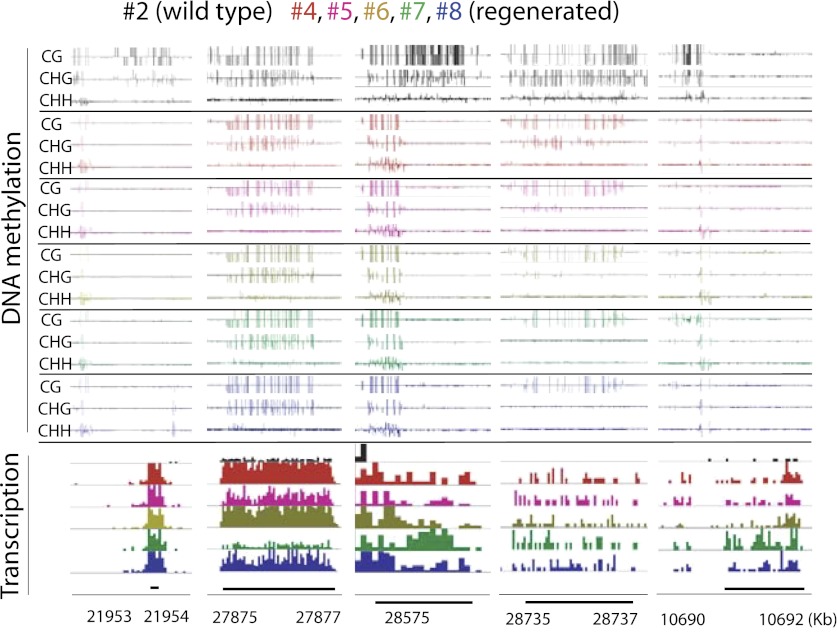
Genome browser views of DNA methylation and gene expression levels. **DOI:** http://dx.doi.org/10.7554/eLife.00354.012

**Figure 4—figure supplement 3.**
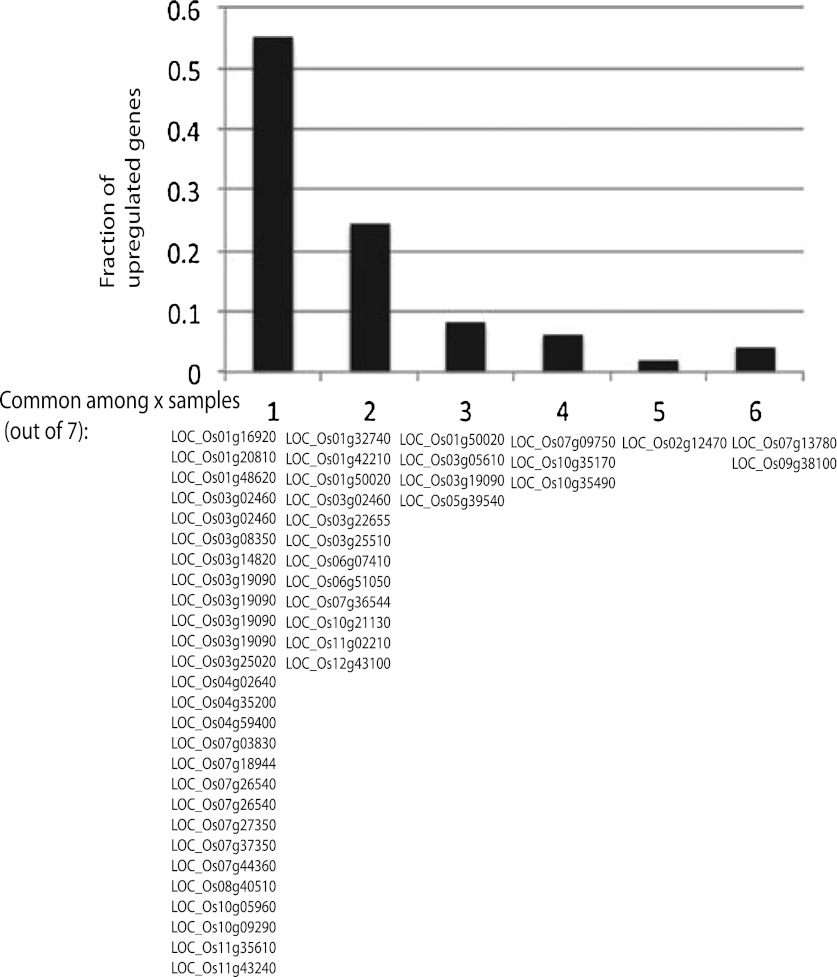
Significantly up-regulated genes are largely different across different lines. Defined significantly up-regulated genes with CG hypomethylation DMRs at promoters were categorized based on the number of lines (out of seven tested) in which they were up-regulated. Gene identifiers are listed below. **DOI:** http://dx.doi.org/10.7554/eLife.00354.013

**Figure 4—figure supplement 4.**
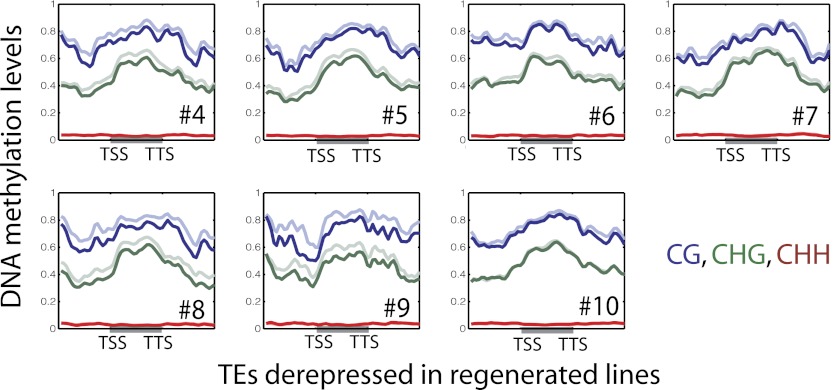
DNA methylation levels over upregulated TE genes in regenerated samples. Average distributions of DNA methylation in wild type (faded lines) and regenerated lines (solid lines) over defined up-regulated TE genes in the indicated regenerated samples. **DOI:** http://dx.doi.org/10.7554/eLife.00354.014

## Additional files

### Major datasets

The following dataset was generated:

StroudH, FengS, JacobsenSE, SimonSA, MeyersBC, 2013, Plants regenerated from tissue culture contain stable epigenome changes in rice, ; http://www.ncbi.nlm.nih.gov/geo/query/acc.cgi?acc=GSE42410, In the public domain at GEO: http://www.ncbi.nlm.nih.gov/geo/.
